# Supplementary material for: Fusion protein strategies for cryo-EM study of G protein-coupled receptors
Source: Nat Commun. 2022 Jul 28;13:4366. doi: 10.1038/s41467-022-32125-2 (PMC9334595; doi:10.1038/s41467-022-32125-2)
Supplement: Supplementary file 1 — Supplementary Information [file 41467_2022_32125_MOESM1_ESM.pdf]

Supplementary Information

Fusion protein strategies for cryo-EM study of G protein-coupled receptors

Kaihua Zhang[1], Hao Wu[1#], Nicholas Hoppe[2#], Aashish Manglik[2,3*] and Yifan Cheng[1,4*]

# These authors contributed equally to this work.
* Corresponding author. Email: Aashish.Manglik@ucsf.edu; Yifan.Cheng@ucsf.edu

This file includes:

Supplementary Figures 1–10

Supplementary Table 1

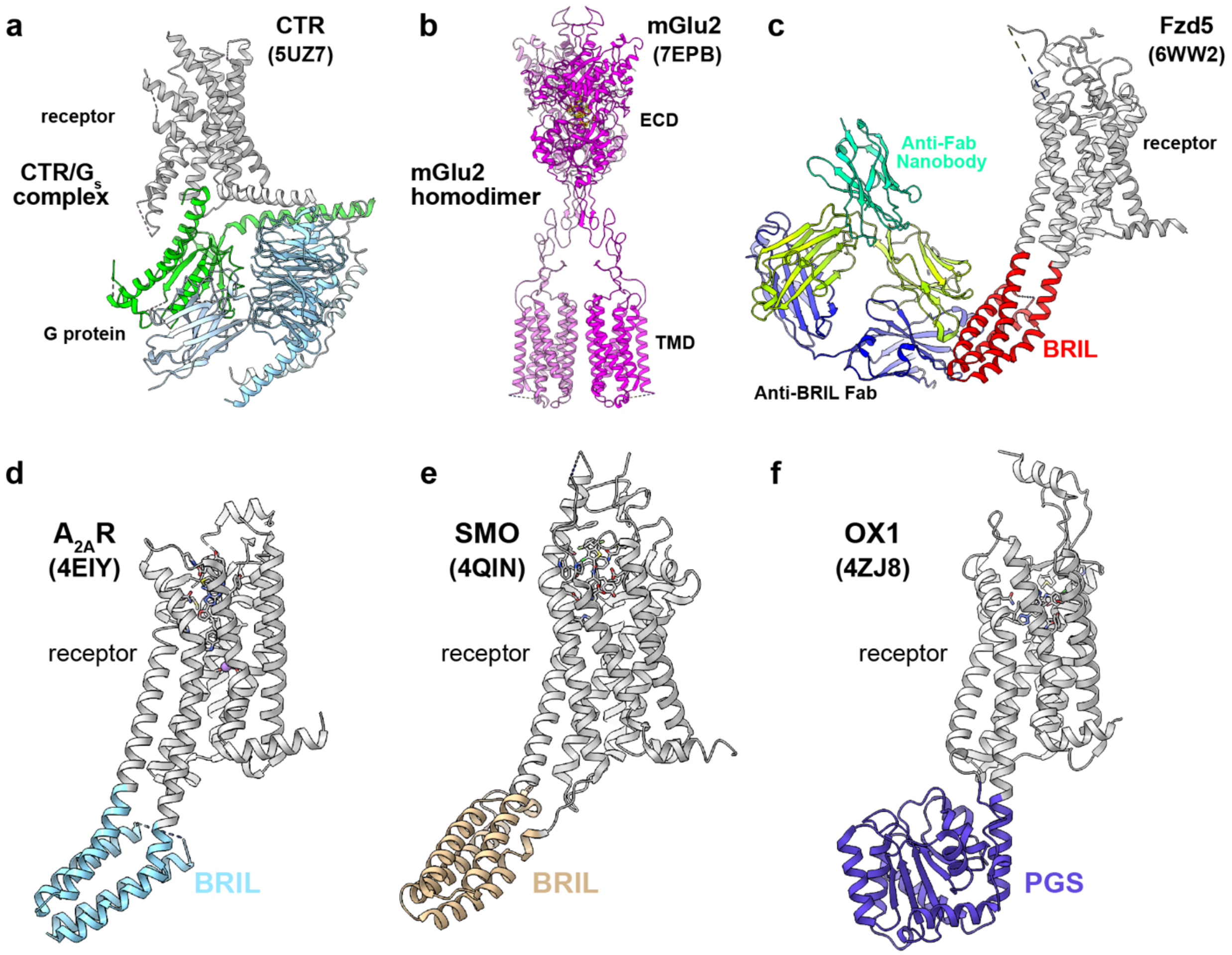

Supplementary Figure 1 | Strategies for structural determination of GPCRs by single particle cryo-EM

a, Calcitonin receptor (CTR)-G_s complex[38] is an example of GPCR/G protein complexes determined by single particle cryo-EM. **b**, Structure of metabotropic glutamate receptors[9] as an example of GPCR dimer determined by single particle cryo-EM. **c**, The cryo-EM structure of hFzd5-Bril in complex with anti-BRIL Fab and anti-Fab nanobody[13]. **d**, **e**, and **f**, Examples of fusion protein BRIL and PGS inserted into A_{2A}R-BRIL[24] (d), SMO[19] (e) and OX1[29] (f). These constructs facilitated structure determination by X-ray crystallography. In these examples, BRIL is inserted to A_{2A}R with two extended helixes (d), to SMO with a single extended helix (e). PGS is inserted with a single extended helix (f).

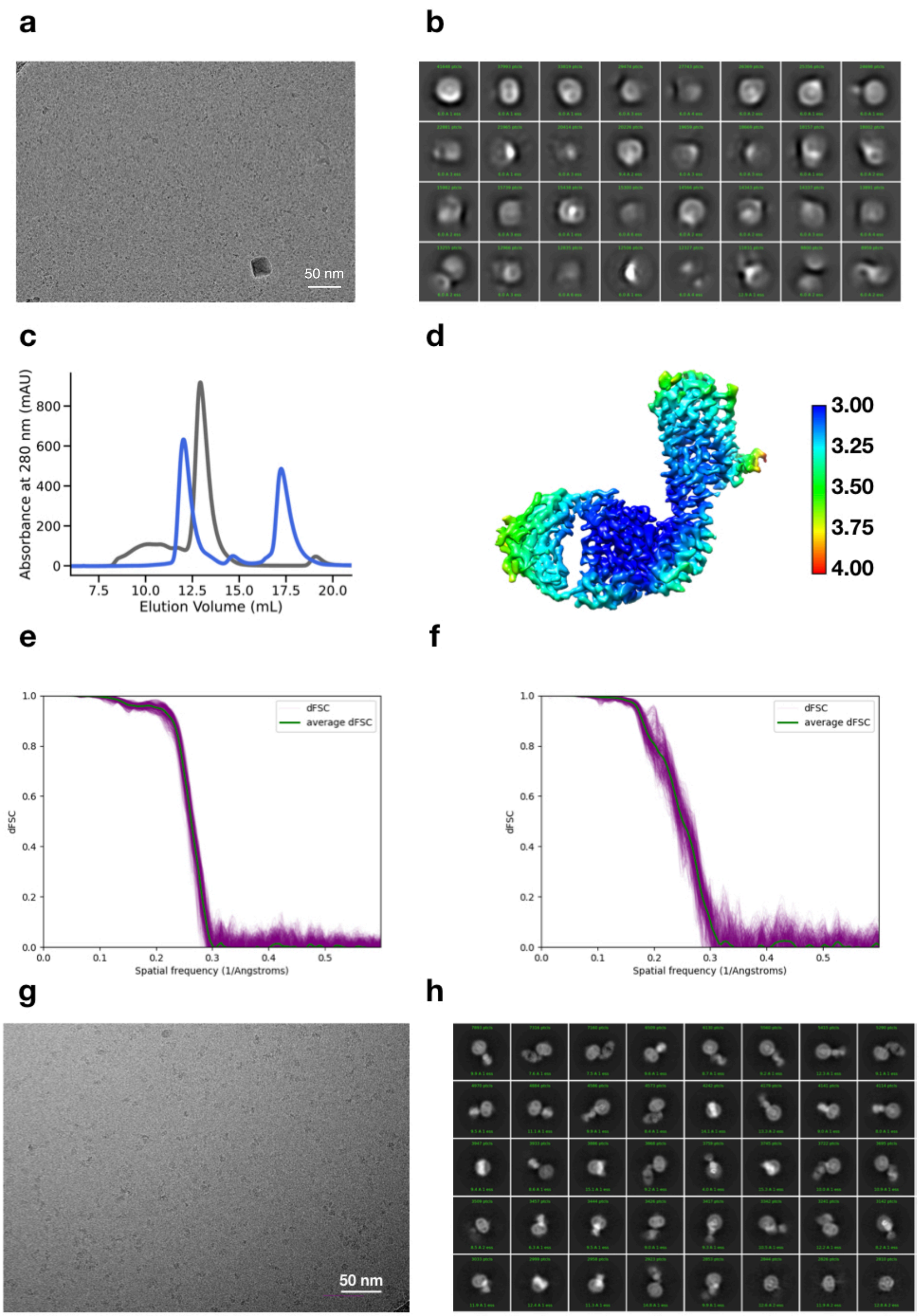

Supplementary Figure 2 | Single particle analysis on A_{2A}R-BRIL

a, Representative raw image of the A_{2A}R-BRIL sample. **b**, 2D average of the A_{2A}R-BRIL sample. **c**, SEC curve of purified A_{2A}R-BRIL (gray) and A_{2A}R-BRIL/Fab complex (blue). **d,** Density map colored by local resolution. **e** and **f**, Directional Fourier shell correlation (dFSC) curves of the intact complex and the TM region, respectively. **g**, Representative raw image of the A_{2A}R-BRIL/Fab complex. **h**, 2D average result of the A_{2A}R-BRIL/Fab complex.

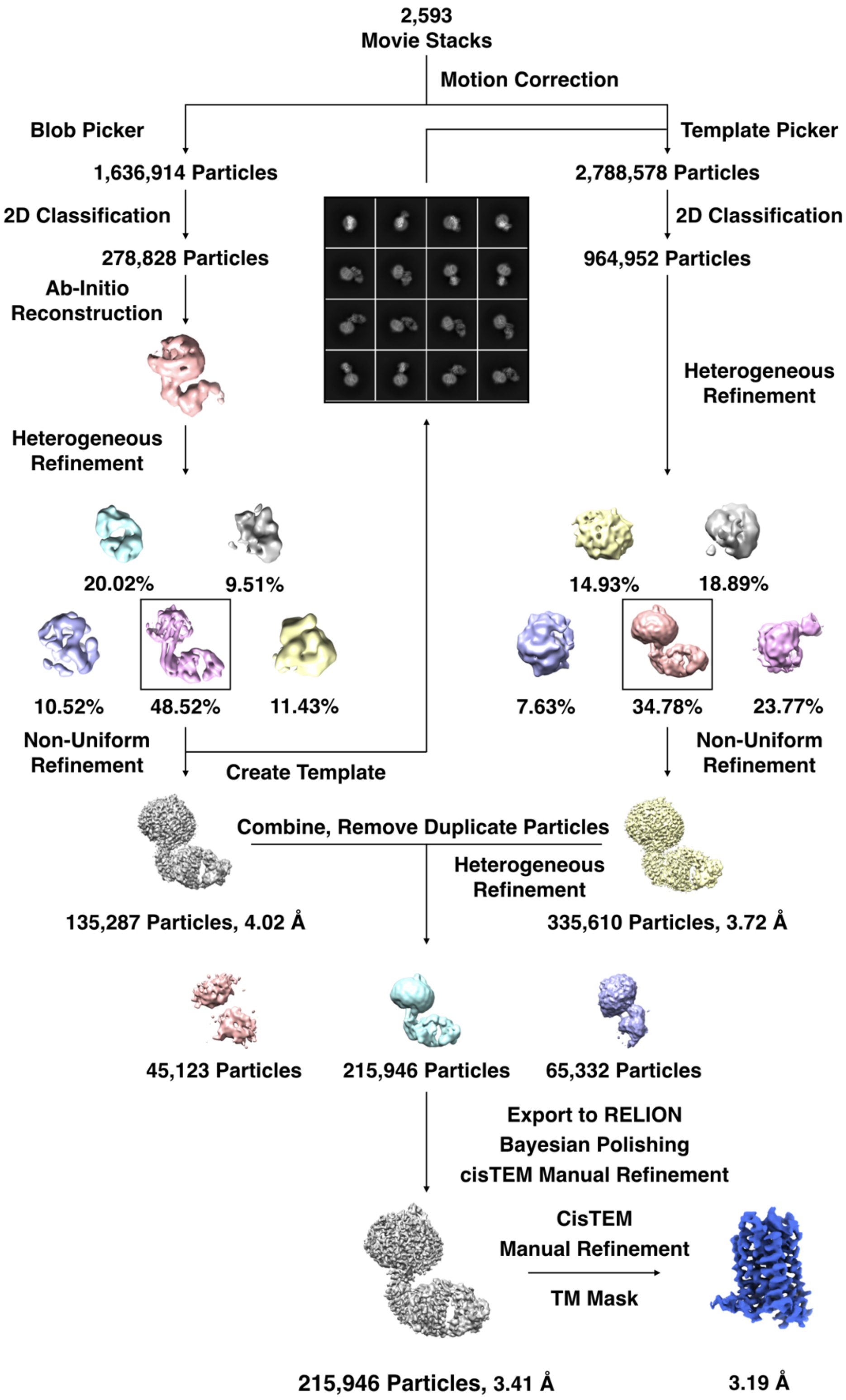

Supplementary Figure 3 | Flow chart of cryo-EM data analysis on A_{2A}R-BRIL/Fab
Flow chart illustrates data processing procedure in determining cryo-EM structure of A_{2A}R-BRIL/Fab complex.

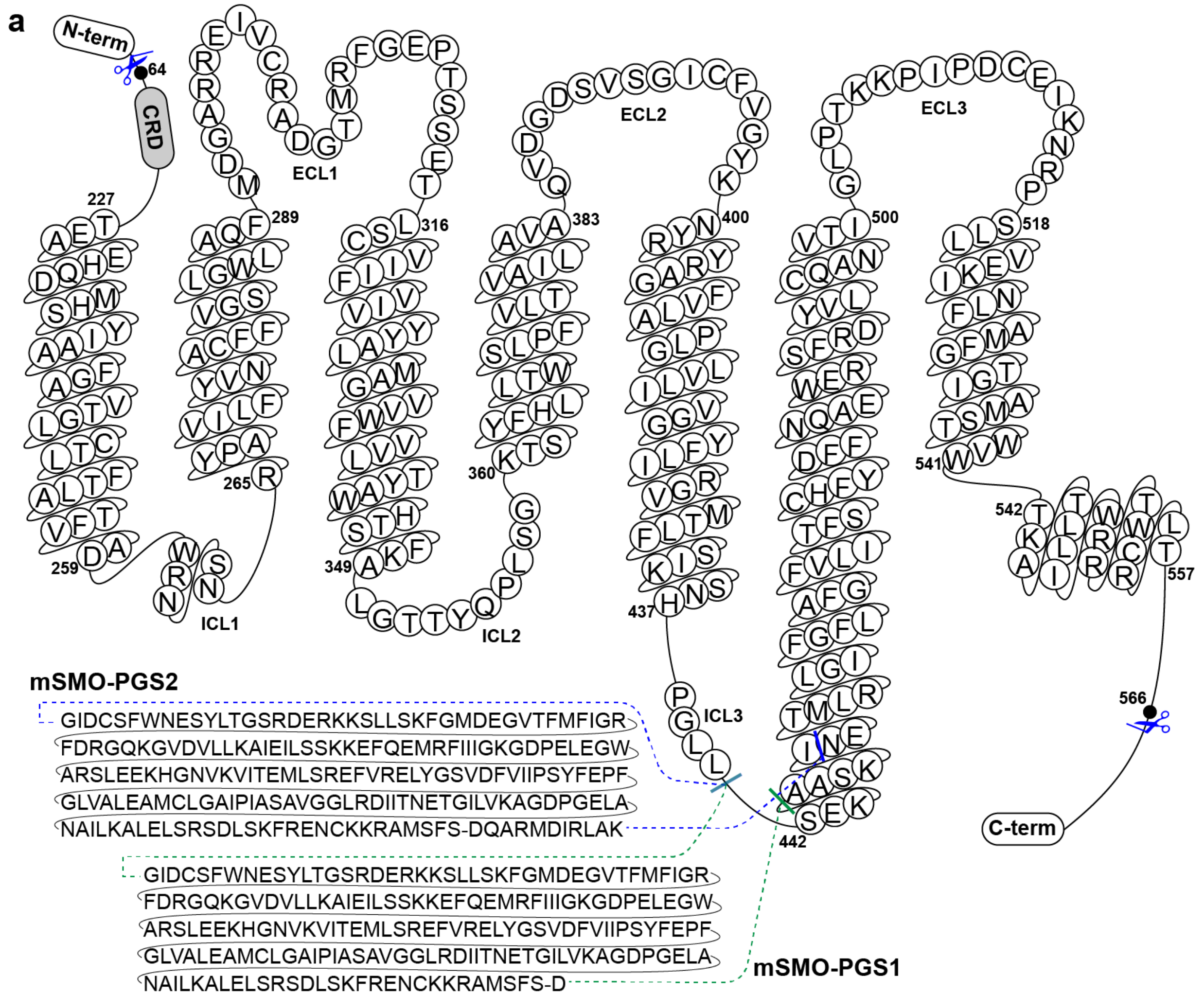

b

		TM5 — ICL3 — TM6	
4O9R/4QIM/4QIN(human)	**414**	VGGYFLIRGVMTLFSIKSNH-------AASKINETMLRLGIFGFLAFGFV	**463**
4JKV/4N4W/6OT0/6XBM(K/J/L)(human)	**414**	VGGYFLIRGVMTLFSIKSNHPGLLSEKAASKINETMLRLGIFGFLAFGFV	**463**
5L7D/5L7I(human)	**414**	VGGYFLIRGVMTLFS-------------SKINETMLRLGIFGFLAFGFV	**463**
5V56/5V57(human)	**414**	VGGYFLIRGVMTLFSIKSNH----------KINETMLRLGIFGFLAFGFV	**463**
6O3C(mouse)	**418**	VGGYFLIRGVMTLFSIKSNHPGLLSEKAASKINETMLRLGIFGFLAFGFV	**467**
6D32/6D35(Xenopus)	**387**	GGGYFLIRGVMTLFS--------------SKINETMLRLGIFGFLAFGFV	**436**
This study_mSMO-PGS1	**418**	VGGYFLIRGVMTLFSIKSNHPGLL---AASKINETMLRLGIFGFLAFGFV	**467**
This study_mSMO-PGS2	**418**	VGGYFLIRGVMTLFSIKSNHPGLL--------NETMLRLGIFGFLAFGFV	**467**

Supplementary Figure 4 | Construct design of SMO-PGS

a, Detailed illustration of two constructs designed for SMO-PGS. **b**, Sequence alignment concerning the modifications of ICL3 in this study and other publications. The construct of mSMO-BRIL in this study has the same insertion of BRIL into ICL3 as used in the crystallization (PDB ID: 4QIN).

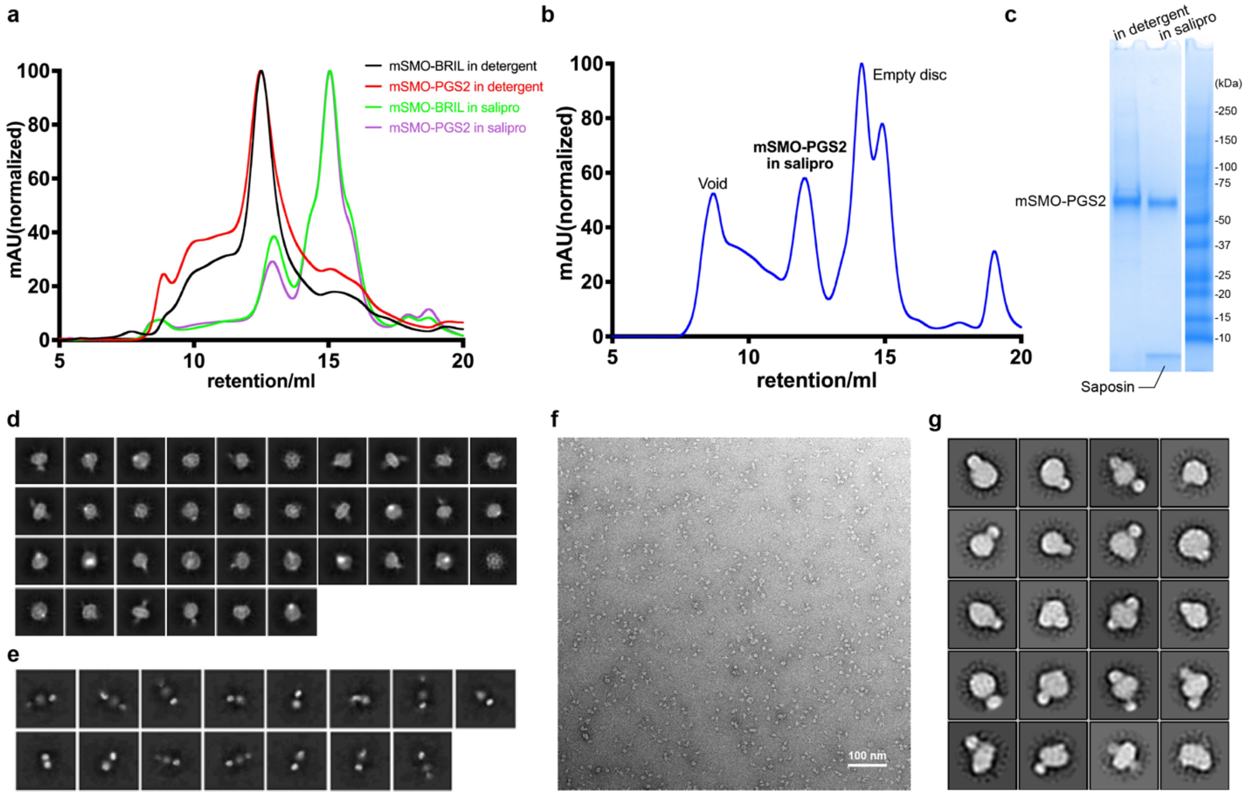

Supplementary Figure 5 | Assessment of SMO-PGS/BRIL preparation

a, Screening results of the purified mSMO-BRIL and mSMO-PGS2 in detergent or salipro by gel filtration. The SEC profile was prepared by the software Graphpad Prism. **b**, Gel filtration result of mSMO-PGS2 in salipro right before cryo grids preparation. **c**, SDS-PAGE gel image stained by Coomassie blue. **d** and **e**, 2D averages of mSMO-BRIL without (d) or with (e) anti-BRIL fab through cryo-EM. The box size is 214 Å for mSMO-BRIL and 412 Å for mSMO-BRIL/anti-BRIL Fab. **f** and **g**, One representative raw micrograph (f) and 2D averages (g) of mSMO-PGS2 through negative-stain EM.

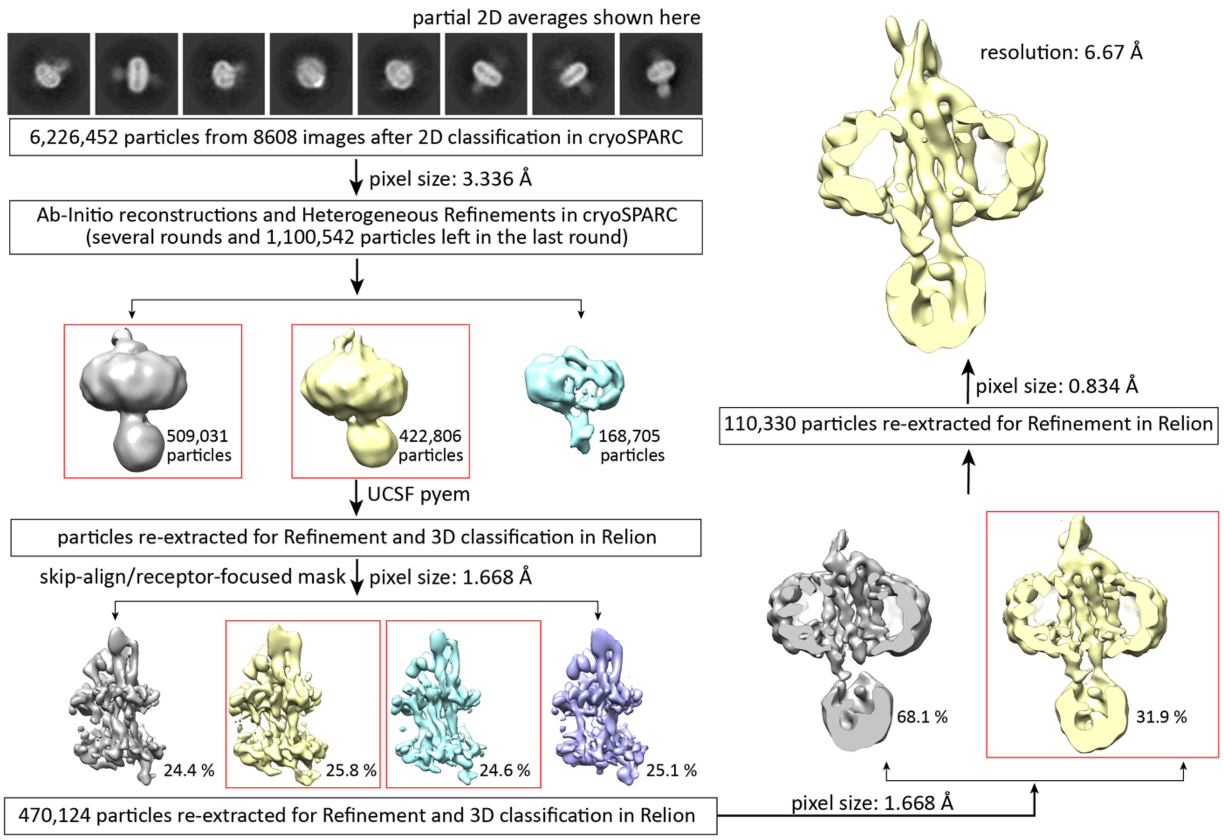

Supplementary Figure 6 | Workflow for the data processing of the construct mSMO-PGS1
Flow chart illustrates data processing procedure in determining cryo-EM structure of mSMO-PGS1.

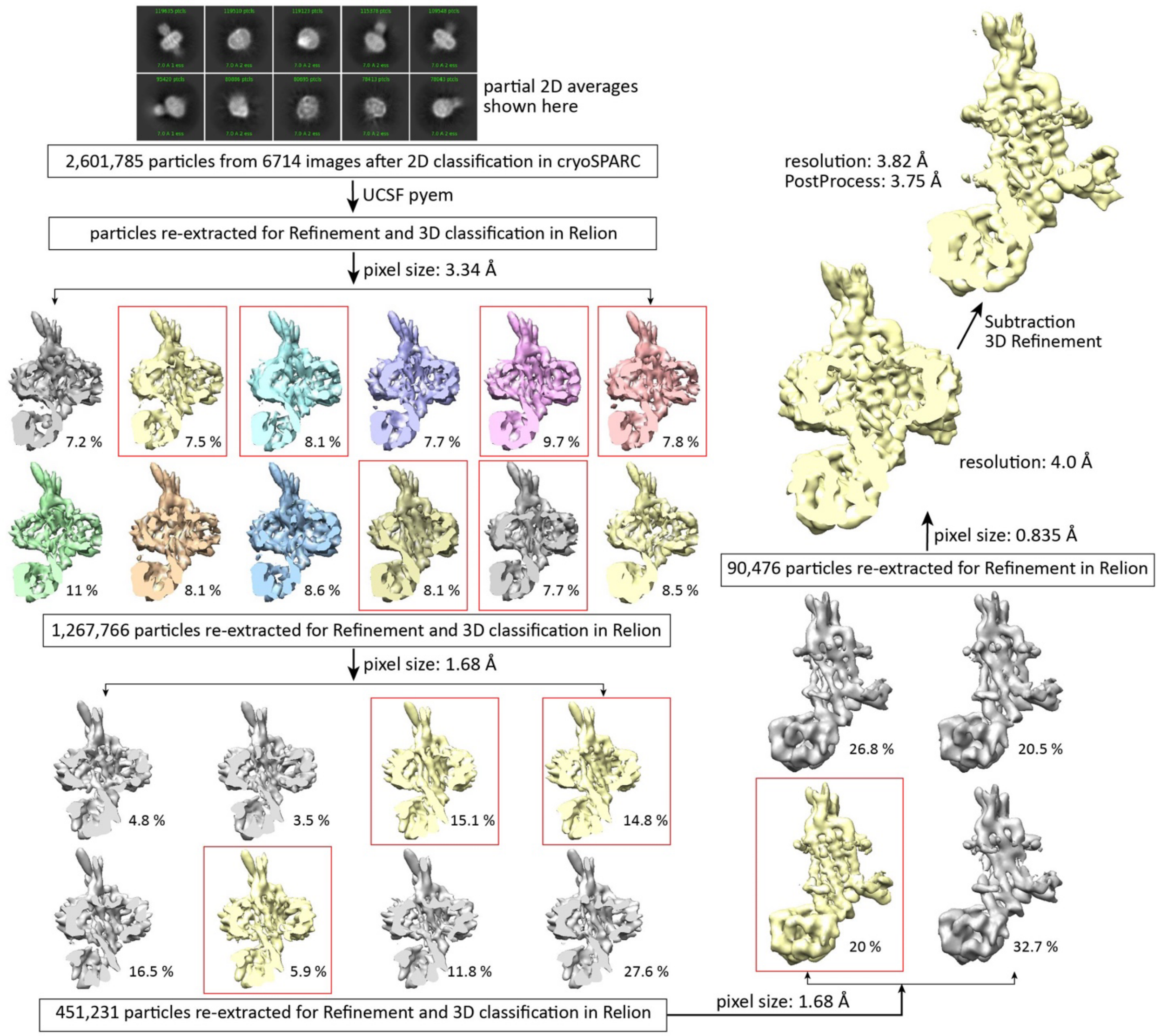

Supplementary Figure 7 | Workflow for the data processing of the construct mSMO-PGS2
Flow chart illustrates data processing procedure in determining cryo-EM structure of mSMO-PGS2.

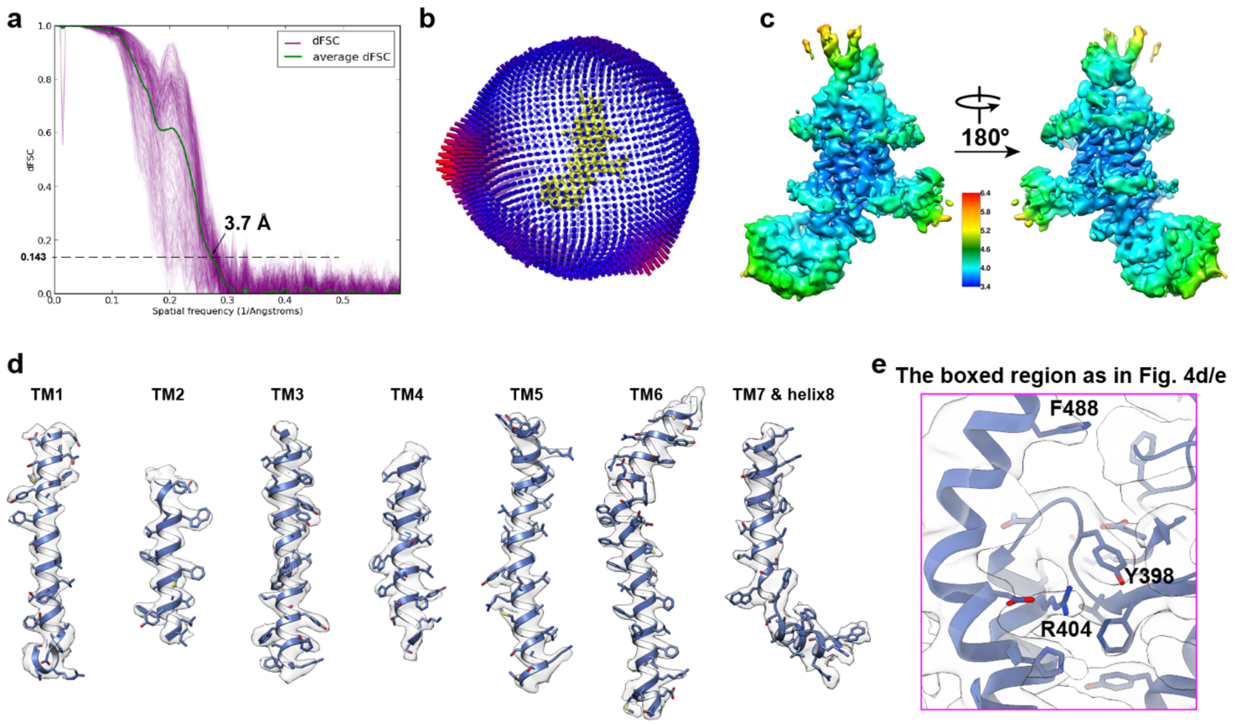

Supplementary Figure 8 | Cryo-EM structure of mSMO-PGS2 at high resolution

a, Directional Fourier shell correlation (dFSC) curves. **b**, Histogram representation of the Euler angle distribution from the final particles used in the reconstruction. **c**, EM map colored using local resolution (Å) information from highest resolution (dark blue) to lowest resolution (red). **d**, Density and atomic model for the transmembrane helices. **e**, The enlarged view of the boxed region as presented in the Figure 4d/e to highlight the modeling of mSMO-PGS2 into the density map. Three residues are labeled including Y398, R404 and F488.

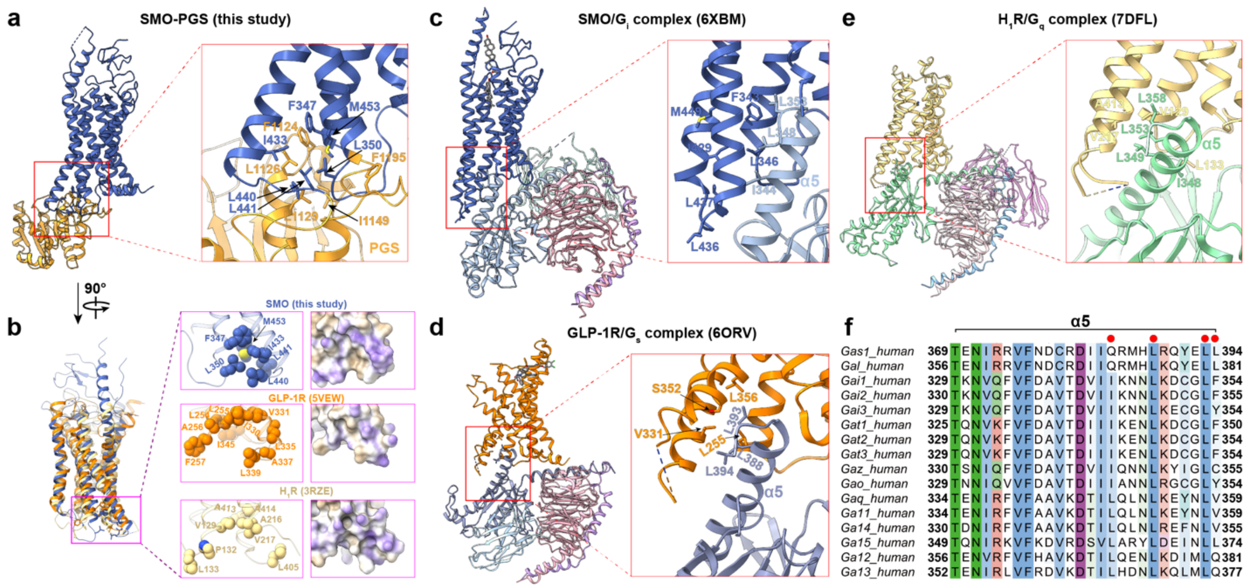

Supplementary Figure 9 | Hydrophobic interactions present in SMO-PGS and GPCR/G protein complexes

a, The hydrophobic interactions between SMO and PGS. **b**, Structural overlay of SMO (this study) with GLP-1R (PDB ID: 5VEW) and H1R (PDB ID: 3RZE). The enlarged views show the key hydrophobic residues, which are labeled and displayed as sphere, and the hydrophobicity surface. **c**, The hydrophobic interactions of human SMO/G_i protein complex. **d**, The hydrophobic interactions of GLP-1R/G_s protein complex. **e**, The hydrophobic interactions of H_1R-G_q protein complex. **f**, Sequence alignment of α5 in G protein family.

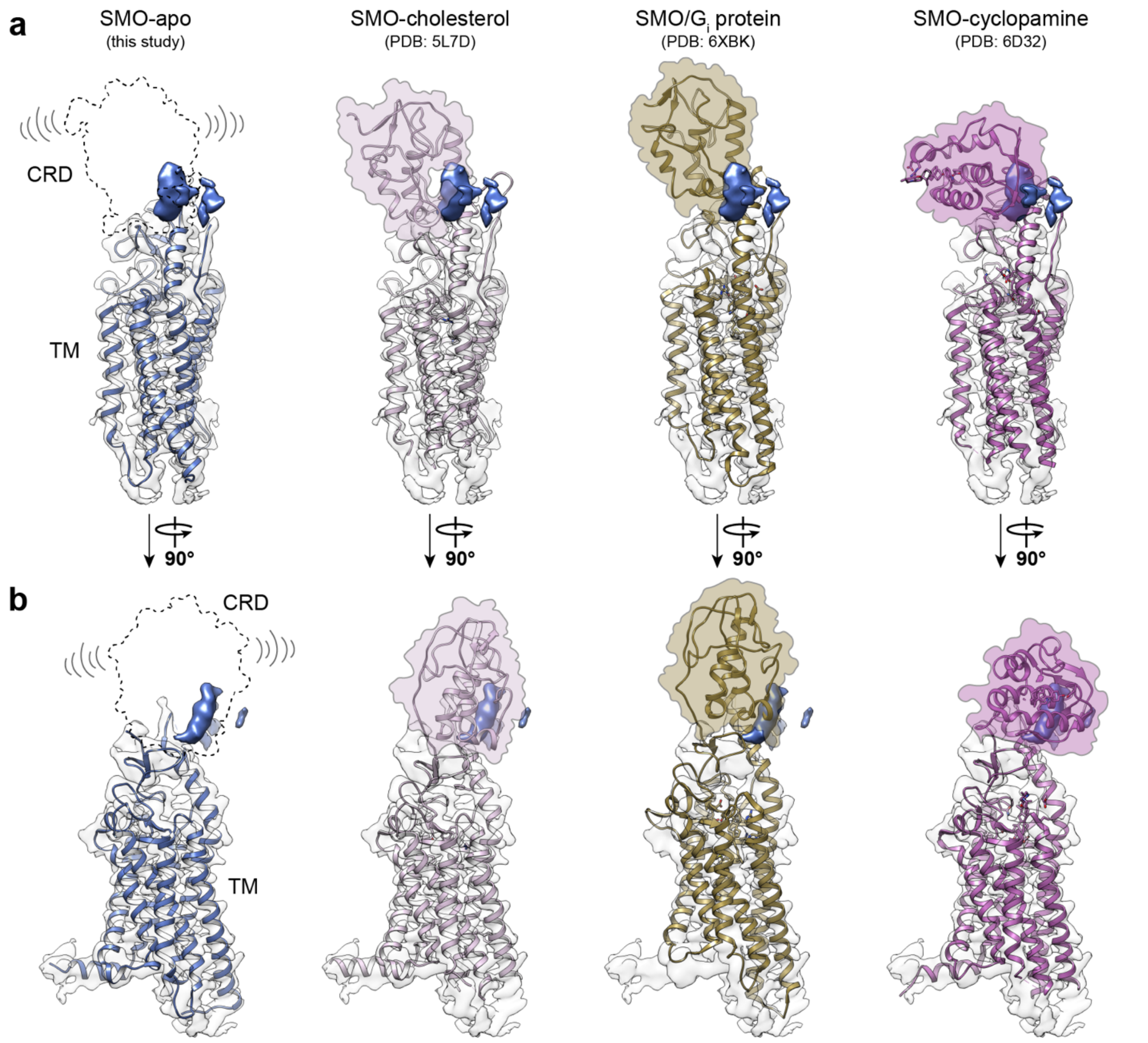

Supplementary Figure 10 | Docking of representative atomic models into mSMO-PGS2 density map

a and **b**, From left to right, docking of de novo built atomic model, SMO-cholesterol, SMO/G$_i$ protein, and SMO-cyclopamine models.

Supplementary Table 1 | Cryo-EM data collection, image processing, and validation statistics

	**mSMO-PGS1 (EMD-27063) no model**	**mSMO-PGS2 (EMD-27062) (PDB-8CXO)**	**A_{2A}R-BRIL/Fab (EMD-25648) (PDB-7T32)**
**Data Collection/Processing**			
Microscope	Titan Krios	Titan Krios	Titan Krios
Detector	Gatan K3	Gatan K3	Gatan K3
Voltage (kV)	300	300	300
Magnification	105,000	105,000	105,000
Defocus Range (μm)	-0.5 to -3.0	-0.5 to -3.0	-1.0 to -2.0
Pixel Size (Å)	0.834	0.835	0.835
Total Electron Dose (e^-/Å^2)	66	68.4	67
Exposure Time (s)	6	6	6
Number of Images	8608	6714	2593
Number of Frames/Image	120	120	117
Symmetry imposed	C1	C1	C1
Initial Particle Number	6,226,452	2,601,785	2,788,578
Final Particle Number	110,330	90,476	215,946
Map resolution (Å)	6.7	3.7	3.4
FSC threshold	0.143	0.143	0.143
**Refinement**			
Initial model used (PDB code)	n/a	6O3C	4EIY
Model resolution (Å)	n/a	4.2	4.1
FSC threshold	n/a	0.5	0.5
Map sharpening B factor (Å^2)	n/a	-165	-100
Model composition			
Non-hydrogen atoms	n/a	4392	2981
Protein residues	n/a	551	390
water	n/a	0	0
Ligands	n/a	1	1
B factors (Å^2)			
Protein	n/a	163	126.92
Ligand	n/a	0	127.55
water	n/a	n/a	n/a
R.m.s. deviations			
Bond lengths (Å)	n/a	0.004	0.0006
Bond angles (°)	n/a	1.017	1.287
Validation			
MolProbity score	n/a	1.09	1.66
Clashscore	n/a	1.6	3.87
Poor rotamers (%)	n/a	0	0
Ramachandran plot			
Favored (%)	n/a	96.89	94.01
Allowed (%)	n/a	2.74	5.21
Disallowed (%)	n/a	0.37	0.78
