# Supplementary material for: Fusion protein strategies for cryo-EM study of G protein-coupled receptors
Source: Nat Commun. 2022 Jul 28;13:4366. doi: 10.1038/s41467-022-32125-2 (PMC9334595; doi:10.1038/s41467-022-32125-2)
Supplement: Supplementary file 3 — Reporting Summary [file 41467_2022_32125_MOESM3_ESM.pdf]

nature portfolio

Corresponding author(s): Aashish Manglik and Yifan Cheng

Last updated by author(s): Jun 27, 2022

Reporting Summary

Nature Portfolio wishes to improve the reproducibility of the work that we publish. This form provides structure for consistency and transparency in reporting. For further information on Nature Portfolio policies, see our Editorial Policies and the Editorial Policy Checklist.

Statistics

For all statistical analyses, confirm that the following items are present in the figure legend, table legend, main text, or Methods section.

n/a	Confirmed	
☐	☒	The exact sample size (*n*) for each experimental group/condition, given as a discrete number and unit of measurement
☒	☐	A statement on whether measurements were taken from distinct samples or whether the same sample was measured repeatedly
☒	☐	The statistical test(s) used AND whether they are one- or two-sided<br>*Only common tests should be described solely by name; describe more complex techniques in the Methods section.*
☒	☐	A description of all covariates tested
☒	☐	A description of any assumptions or corrections, such as tests of normality and adjustment for multiple comparisons
☒	☐	A full description of the statistical parameters including central tendency (e.g. means) or other basic estimates (e.g. regression coefficient) AND variation (e.g. standard deviation) or associated estimates of uncertainty (e.g. confidence intervals)
☒	☐	For null hypothesis testing, the test statistic (e.g. *F*, *t*, *r*) with confidence intervals, effect sizes, degrees of freedom and *P* value noted<br>*Give P values as exact values whenever suitable.*
☒	☐	For Bayesian analysis, information on the choice of priors and Markov chain Monte Carlo settings
☒	☐	For hierarchical and complex designs, identification of the appropriate level for tests and full reporting of outcomes
☒	☐	Estimates of effect sizes (e.g. Cohen's *d*, Pearson's *r*), indicating how they were calculated

Our web collection on statistics for biologists contains articles on many of the points above.

Software and code

Policy information about availability of computer code

Data collection	EM images are acquired using SerialEM 3.8, followed by on-the-fly motion correction by MotionCor 2 1.3.1.
Data analysis	Image and map analyses were performed using these softwares: MotionCor 2 1.3.1, Relion 3.1, CryoSPARC 3.1.0, UCSF Chimera 1.14, UCSF ChimeraX 1.3, Graphpad Prism 8, PHENIX 1.19, COOT 0.9.4 and pyem 0.5. Protein sequences were aligned using Clustal Omega.

For manuscripts utilizing custom algorithms or software that are central to the research but not yet described in published literature, software must be made available to editors and reviewers. We strongly encourage code deposition in a community repository (e.g. GitHub). See the Nature Portfolio guidelines for submitting code & software for further information.

Data

Policy information about availability of data

All manuscripts must include a data availability statement. This statement should provide the following information, where applicable:

- Accession codes, unique identifiers, or web links for publicly available datasets
- A description of any restrictions on data availability
- For clinical datasets or third party data, please ensure that the statement adheres to our policy

EM density maps and the related coordinates are deposited to the Electron Microscopy Data Bank (EMDB) and Protein Data Bank (PDB) with access code EMD-25648 and 7T32 (A2AR-BRIL/Fab), EMD-27063 (mSMO-PGS1), EMD-27062 and 8CXO (mSMO-PGS2). All other data are available from corresponding authors upon reasonable request.

Human research participants

Policy information about studies involving human research participants and Sex and Gender in Research.

Reporting on sex and gender	*Use the terms sex (biological attribute) and gender (shaped by social and cultural circumstances) carefully in order to avoid confusing both terms. Indicate if findings apply to only one sex or gender; describe whether sex and gender were considered in study design whether sex and/or gender was determined based on self-reporting or assigned and methods used. Provide in the source data disaggregated sex and gender data where this information has been collected, and consent has been obtained for sharing of individual-level data; provide overall numbers in this Reporting Summary. Please state if this information has not been collected. Report sex- and gender-based analyses where performed, justify reasons for lack of sex- and gender-based analysis.*
Population characteristics	*Describe the covariate-relevant population characteristics of the human research participants (e.g. age, genotypic information, past and current diagnosis and treatment categories). If you filled out the behavioural & social sciences study design questions and have nothing to add here, write "See above."*
Recruitment	*Describe how participants were recruited. Outline any potential self-selection bias or other biases that may be present and how these are likely to impact results.*
Ethics oversight	*Identify the organization(s) that approved the study protocol.*

Note that full information on the approval of the study protocol must also be provided in the manuscript.

Field-specific reporting

Please select the one below that is the best fit for your research. If you are not sure, read the appropriate sections before making your selection.

☒ Life sciences ☐ Behavioural & social sciences ☐ Ecological, evolutionary & environmental sciences

For a reference copy of the document with all sections, see nature.com/documents/nr-reporting-summary-flat.pdf

Life sciences study design

All studies must disclose on these points even when the disclosure is negative.

Sample size	Each data set contains sufficient number of original micrographs (movie stacks), from which over a million raw particles were selected using automated particle picking program. A total of three dataset were collected to determine three structures in this study. The number of micrographs and particles in each dataset is presented in Supplementary Table 1.
Data exclusions	No data were excluded.
Replication	No replication in data acquisition, which is not required for structural studies.
Randomization	Single particle cryo-EM data processing follows gold standard, that is to separate particle stacks by the order, even or odd, into two separate data sets, and processed separately. This separation is considered random. All biochemical experiments were initiated from multiple independent aliquots of the frozen cells expressing the target proteins, and then subjected to indicated experimental conditions.
Blinding	All experiments are not blind. In structural biology, it is typical the investigators know the target of the study. Blindness is not standard procedure in structural studies.

Reporting for specific materials, systems and methods

We require information from authors about some types of materials, experimental systems and methods used in many studies. Here, indicate whether each material, system or method listed is relevant to your study. If you are not sure if a list item applies to your research, read the appropriate section before selecting a response.

Materials & experimental systems

n/a	Involved in the study
	[x] Antibodies
	[x] Eukaryotic cell lines
[x]	Palaeontology and archaeology
[x]	Animals and other organisms
[x]	Clinical data
[x]	Dual use research of concern

Methods

n/a	Involved in the study
[x]	ChIP-seq
[x]	Flow cytometry
[x]	MRI-based neuroimaging

Antibodies

Antibodies used	Fab against the fusion protein BRIL (apocytochrome b562)
Validation	The vector containing the Fab gene is the gift from Kossiakoff lab (https://www.nature.com/articles/s41467-020-15363-0)

Eukaryotic cell lines

Policy information about cell lines and Sex and Gender in Research

Cell line source(s)	Human embryonic kidney (HEK293 GnTi) purchased from ATCC, Expi293F cells purchased from Thermo Fisher and Sf9 Insect Cells purchased from Expression Systems.
Authentication	HEK293 cells, Expi293 cells and Insect cells were directly purchased from vendors, where they were validated. None of the cell lines were authenticated by the authors. SDS-PAGE results and EM reconstructions confirmed the expected A2AR and SMO protein production from the cells.
Mycoplasma contamination	Cell lines were certified as testing negative for Mycoplasma by the vendors. All cell lines exhibited normal growth pattern. All cells were cultured in a dedicated incubator free from potential contaminating primary or secondary cultures, and were used for expression of receptors and biochemical and cryo-EM studies.
Commonly misidentified lines (See ICLAC register)	None.
